# Veterinary College Notes

**Published:** 1890-11

**Authors:** 


### VETERINARY COLLEGE NOTES.

*Department of Veterinary Medicine, University of Minnesota.*—This new school of Veterinary Medicine will open its course for students of the first and second year on October 8th. Dr. Frank Allen, D. V. S., has been appointed instructor of Anatomy, Dr. S. D. Brimball instructor in Surgery. The course has been lengthened from six to eight months in continued session. An addition to the Veterinary Hospital in St. Anthony Park has been made for the reception of small animals. Dr. Schwartskopff has published an article on the "Influence of Modern Feeding of Pigs upon the Formation of the Skull and Dentition."

*New York College of Veterinary Surgeons.*—The opening address of the present session was given by Professor Searing on the evening of October 1st. Seventy-two regular students attended the first lecture.

*The Baltimore University School of Veterinary Science.*—The session 1890–91 commenced on Wednesday, October 1st, being formally opened on Tuesday evening by the President of the Faculty, Professor Ward, who, in his remarks to the students, said there was a bright future for those who were students in the strict meaning of the sense. The way has been prepared for the educated veterinarian to hold a good position in society through the efforts of the leading teachers and members of the profession, by whose combined efforts the veterinary profession is now looked upon and recognized as one of the several arts and sciences. The attainment of such recognition, however, depended on the earnest exertions of each individual student now during his college training.

About twenty have applied for admission as students, of which number fourteen were present.

The Faculty has leased the premises No. 1513 East Baltimore Street, near the University building. There is accommodation for forty horses, large shoeing forge, and operating box on the ground floor, with three rooms over twenty by forty feet each, to be converted into lecture-room, dissecting-room, museum and students'-room, thus forming a very compact and complete college building.

In consequence of Prof. Wray's removal to England as one of the United States Sanitary Inspectors, some change had to be made in the chairs.

Prof. Ward takes Prof. Wray's chair of Theory and Practice of Veterinary Medicine, and hands over Contagious and Parasitic Diseases to Dr. Albert Haskell, M. R. C. V. S., who will also assist Prof. Cuddy in Materia Medica and Botany.

Prof. Eilan, M. D., has been appointed to the chair of Physiology, in place of Prof. Reid, M. D.

